# Monoclonal Antibodies Directed to Fucoidan Preparations from Brown Algae

**DOI:** 10.1371/journal.pone.0118366

**Published:** 2015-02-18

**Authors:** Thomas A. Torode, Susan E. Marcus, Murielle Jam, Thierry Tonon, Richard S. Blackburn, Cécile Hervé, J. Paul Knox

**Affiliations:** 1 Centre for Plant Sciences, Faculty of Biological Sciences, University of Leeds, Leeds, United Kingdom; 2 Sorbonne Universités, UPMC Univ Paris 06, UMR 8227, Integrative Biology of Marine Models, Station Biologique de Roscoff, CS 90074, Roscoff, France; 3 CNRS, UMR 8227, Integrative Biology of Marine Models, Station Biologique de Roscoff, CS 90074, Roscoff, France; 4 Sustainable Materials Research Group, Centre for Technical Textiles, University of Leeds, Leeds, United Kingdom; University of New South Wales, AUSTRALIA

## Abstract

Cell walls of the brown algae contain a diverse range of polysaccharides with useful bioactivities. The precise structures of the sulfated fucan/fucoidan group of polysaccharides and their roles in generating cell wall architectures and cell properties are not known in detail. Four rat monoclonal antibodies, BAM1 to BAM4, directed to sulfated fucan preparations, have been generated and used to dissect the heterogeneity of brown algal cell wall polysaccharides. BAM1 and BAM4, respectively, bind to a non-sulfated epitope and a sulfated epitope present in the sulfated fucan preparations. BAM2 and BAM3 identified additional distinct epitopes present in the fucoidan preparations. All four epitopes, not yet fully characterised, occur widely within the major brown algal taxonomic groups and show divergent distribution patterns in tissues. The analysis of cell wall extractions and fluorescence imaging reveal differences in the occurrence of the BAM1 to BAM4 epitopes in various tissues of *Fucus vesiculosus*. In *Ectocarpus subulatus*, a species closely related to the brown algal model *Ectocarpus siliculosus*, the BAM4 sulfated epitope was modulated in relation to salinity levels. This new set of monoclonal antibodies will be useful for the dissection of the highly complex and yet poorly resolved sulfated polysaccharides in the brown algae in relation to their ecological and economic significance.

## Introduction

Brown algae are a large and diverse class of organisms which dominate most temperate coastal environments. They fulfil important roles as primary producers within intertidal zones, and are key ecological players in some marine environments by being the first recruited species for the organization and structuring of ecosystems. Brown algae vary in size from small filamentous species such as those belonging to the Ectocarpales which can be grown *in vitro* in laboratories, to giant kelps of the Laminariales which can reach 60 m in length [1]. Previous research, including studies on early embryogenesis, has focused on species of the Fucales, which grow in the intertidal regions of most coasts in the northern hemisphere [2]. More recently, the development of the filamentous *Ectocarpus siliculosus* as a genetic model organism for brown algae [3] has paved the way for studies on different aspects of brown algal biology including early morphogenesis and life cycles [4,5], response to abiotic change [6] and evolution of species [7,8]. Furthermore, the divergent evolution of brown algae when compared to plants and animals has led to unique biochemical pathways resulting in a range of novel bioactive compounds and polymers including those in cell walls [9]. Hence brown algae have received a renewed interest as a source of biomass that does not compete with arable land. Indeed, brown algal polymers have been used in high-capacity lithium ion batteries [10], to produce nanoparticles with enhanced delivery efficiency for gene and drug delivery [11] in addition to processes for the production of ethanol [12–14].

Brown algal cell walls are composed predominantly of polysaccharides together with lower amounts of phenolic substances, proteins and halide compounds such as iodide. The polyanionic polysaccharides alginates and sulfated fucans are prevalent over neutral and crystalline polysaccharides including cellulose [15]. Alginates are linear polymers of two 1,4-linked uronic acids: β-d-mannuronic acid and α-l-guluronic acid [16]. Sulfated fucans or fucoidans are collective terms that group a highly diverse spectrum of sulfated polysaccharides containing α-l-fucose residues. They can generally be divided into homopolymers called homofucans or heteropolymers [9,15–19]. Backbones of homofucans are invariably made of 1,3- or 1,3–1,4-linked α-l-fucose, while backbones of heterofucans are more diverse and can be based on neutral sugars and/or uronic acid residues (i.e. glycuronofucogalactans, xylofucoglycuronans, fucomannoglucuronans) [16,20,21]. The fucose residues are commonly sulfated at positions 2, 3 and/or 4. Alternatively they can be substituted by methyl or acetyl groups, or branched with additional fucose, xylose or uronic acid residues. Some prokaryotes and most eukaryotic organisms produce sulfated carbohydrates, and this ability is likely to be of ancestral origin [9,22]. Exceptions are the freshwater and land plants which have probably lost such an ability or requirement during the conquest of land, as a functional adaptation to sulfate-depleted habitats. Marine angiosperms however do produce sulfated polysaccharides as a result of their secondary exploitation of marine environments and polysaccharide sulfation is positively correlated with increasing saline conditions [23–25]. In the green macroalgae *Lamprothamnium papulosum*, the extracellular sulfated mucilage increases in thickness and sulfate content with increasing salinity [24,25]. In brown algae the concentrations of sulfate groups on fucans positively correlate with increasing exposure to the atmosphere in the intertidal zone, suggesting a role in desiccation resistance [26]. A strain of the species *Ectocarpus subulatus*, isolated from a true freshwater environment [27] undergoes major morphological, transcriptomic and metabolic changes under variable salinities, including alteration of the expression of genes encoding enzymes potentially involved in the sulfation or de-sulfation of cell wall polysaccharides [28].

Sulfated fucans have been studied extensively due to their numerous therapeutic effects and bioactivities [18,19,29]. Understanding their chemistry and heterogeneity is critical in developing methods to extract and isolate sulfated fucans for applications. Separation of fucans into various fractions has been achieved [30] but it still remains unclear how sulfated fucans vary within cell walls and what are the structural bases of any linkages or interactions that may exist with other polymers such as cellulose or alternatively alginates [15]. Differences between species and extraction techniques [31] hinder our understanding of the sulfated fucans and their heterogeneities. Monoclonal antibodies, with their versatility of applications for both *in situ* and quantitative analysis, are useful to complement physicochemical analyses. Previously, monoclonal antibodies to brown algal cell wall polymers have been generated but not characterized in relation to sulfate patterning [32–35]. In order to study the structure and sulfate patterning of brown algal fucans, and their relation to biological functions, we have generated four novel monoclonal antibodies that bind to sulfated fucan/fucoidan preparations. These probes are used in assays and procedures to dissect the heterogeneity of sulfated fucan preparations and of brown algal cell walls.

## Materials and Methods

### Animals

The use of rats to generate monoclonal antibodies was carried out in strict accordance with the guidelines and licence of United Kingdom Home Office under Project Licence PL4003426 / Animals (Scientific Procedures) Act 1986. Procedures included injection and withdrawal of blood and were performed by the same trained staff and all efforts were made to minimize suffering. An inhalational agent (isoflurane) was used for the initial induction and the maintenance of general anaesthesia via an anaesthetic delivery system. The animals were sacrificed by exposure to carbon dioxide gas in a rising concentration which is an approved method listed in the Schedule 1 of the UK Animals (Scientific Procedures) Act 1986.

### Algal samples

Samples of algae, except the *Ectocarpus* samples, were collected from natural environments as follows: Guernsey, Channel Islands GPS coordinates: 49.43–2.66 (*Laminaria digitata*, *Pelvetia canaliculata*); Shetland Islands, UK GPS: 60.42–1.09 (*Chorda filum*); Scarborough, UK GPS: 54.27–0.39 (*Halidrys siliquosa*, *Laminaria hyperborea*, *Saccharina latissima*); Roscoff, France GPS: 48.72–3.98 (*Ascophyllum nodosum*, *Fucus serratus*, *Fucus spiralis*, *Himanthalia elongata*, *Laminaria digitata*, *Laminaria hyperborea*, *Laminaria ochroleuca*, *Saccharina latissima*, *Sargassum muticum*, *Pelvetia canaliculata*, *Undaria pinnatifida*). No permissions were required for these locations/activities. The collection of brown algae samples did not involve endangered or protected species. *Ectocarpus siliculosus* (marine strain, Ec32) and *Ectocarpus subulatus* (freshwater strain, Ec371) were both cultured at Station Biologique de Roscoff as described [28].

### Generation of sulfated fucan-directed rat monoclonal antibodies

Four rat monoclonal antibodies (MAbs), designated BAM1 to BAM4, were derived subsequent to immunisation with a neoglycoprotein immunogen, prepared by coupling of fucoidan (Sigma-Aldrich F5631) to bovine serum albumin (BSA) by activation with 1-cyano-4-dimethylaminopyridium tetrafluroborate (CDAP) [36]. Two male Wistar rats were each injected with 250 μg fucoidan-BSA in complete Freund’s adjuvant administered subcutaneously on day 0, and the same was administered with incomplete Freund’s adjuvant on days 31, 59, 125 and 158. A pre-fusion boost of 100 μg fucoidan-BSA in 1 ml PBS was administered on day 215 prior to spleen removal. Hybridoma production and cloning procedures were performed as described [37].

### Polysaccharides

Fucoidan (F5631), alginate (A7003), laminaran (L9634), carrageenan (C1013), sulfated Dextran (D6001), chondroitin (C4384), oat spelt xylan (X0627), gum Arabic (G9752) and citrus pectin (P9135) were obtained from Sigma-Aldrich. Tamarind xyloglucan, potato galactan, guar galactomannan, sugar beet arabinan and citrus polygalacturonan were obtained from Megazyme International (Bray, Ireland).

### Enzyme-linked immunosorbent assays

ELISAs were performed in 96-well microtitre plates (Maxisorb, NUNC) coated with 100 μL per well of antigen (50 μg/mL) in phosphate-buffered saline (PBS, 137 mM NaCl) overnight at 4°C. Unbound antigen was washed out using tap water, and 200 μL of blocking solution of 5% milk powder in PBS (MP/PBS) were added per well. After 1 h at room temperature (RT), plates were rinsed in tap water and 100 μL of hybridoma supernatant in MP-PBS were added at desired dilution. Unless otherwise stated, BAM MAb hybridoma cell supernatants were used at 25-fold dilution, except for BAM1, which was used at 50-fold dilution to generate equivalent signals. Plates were incubated at RT for 1.5 h, washed with tap water, and then incubated with secondary antibody (rabbit anti-rat IgG, whole molecule, coupled to horseradish peroxidase (HRP), Sigma-Aldrich) at a dilution of 1:1000 for 1.5 h and at RT. Plates were washed in tap water, and antibody binding was detected by the addition of 150 μL per well of HRP substrate (0.1 M sodium acetate buffer, pH 6.0, 1% tetramethyl benzidine, 0.006% (v/v) H_2_O_2_) and allowed to develop for 5 min before the reaction was stopped by the addition of 30 μL 2.5 M H_2_SO_4_.The absorbance at 450 nm was read with a microtitre plate reader. For Azure A inhibition studies, coated and blocked microtitre plates were incubated with 1 mg/ml of Azure A (Sigma-Aldrich) for 1 h, washed, probed with antibodies, and developed as for standard ELISA techniques.

### De-sulfation and sulfate assay

Sulfated fucan fraction FS28 [38] (100 mg) was passed through a Dowex 50-W cation exchange resin (H^+^ 200–400 mesh, Sigma-Aldrich), eluted with water, collected over 20% (v/v) pyridine, dialysed against distilled water (MCWO 6–8 kDa) and then freeze-dried. The pyridine salt was then re-suspended at 1% (w/v) in anhydrous pyridine, and 10% (v/v) total volume of chlorotrimethysilane (CTMS) was added. The solution was incubated at 100°C for 3 h. Samples were taken at different incubation times (DS0, before incubation; DS1 and DS2, at 1 and 2 h into the incubation, respectively). The reaction was ended at sampling time points by dropwise addition of distilled water to destroy excess CTMS. All solutions were then dialysed (MWCO 3.5 kDa) sequentially against running tap water, distilled water, 0.1 M NaCl (to remove any unreactive pyridine salts) and distilled water and freeze-dried. Sulfate concentrations were measured as equivalents of FS28, by colorimetric quantification using Azure A (Sigma). Polysaccharide samples (50 μl, concentrations ranging from 0.3 to 5 mg/ml) were mixed with 50 μl 2.5 M H_2_SO_4_, before addition of 900 μl of Azure A (20 μg/ml) and determination of absorbance at 620 nm in a microtitre plate reader (200 μl/well). Absorbances were measured against a standard curve of FS28 with concentrations ranging from 0 to 1 mg.

### Treatment of glycans with sodium metaperiodate

Assessment of the impact of periodate treatment on antigens followed a published method [39]. Antigen-coated microtitre plates were incubated with 200 μl per well of 25 mM sodium metaperiodate (Sigma-Aldrich) in 50 mM sodium acetate buffer (pH 4.5), or with buffer only for controls, and kept in the dark at 4°C for the required time. Plates were then washed, blocked and developed as for standard ELISA.

### Epitope detection chromatography (EDC)

EDC analysis of sulfated fucans was based on a technique described for plant glycans [40]. Samples (400 μg) in 50 mM sodium acetate buffer pH 5.5 were loaded onto a 1 ml anion-exchange column (Hi Trap, GE Healthcare), and eluted with a gradient 0 to 5 M of NaCl in 50 mM sodium acetate pH 5.0 buffer. Fractions (1 ml) were collected, and neutralised with 50 μl of 1 M sodium carbonate and 100 μl aliquots of fractions were then used to coat microtitre plates, further developed as for standard ELISA.

### Algal materials, cell wall extractions and preparation for microscopy

All samples of algae, except the *Ectocarpus* samples, were collected from natural environments (see statement above). Algae were cleaned of epiphytes and washed in tap water. *Ectocarpus* samples used for the preparation of the alcohol insoluble residues (AIRs) were grown in 10 L flasks, while the algae used for *in situ* fluorescence imaging were grown in 140 mm Petri dishes.

Samples for cell wall extractions were oven-dried, blended finely and exhaustively washed sequentially in 70, 80, 90, 100% ethanol, acetone, and chloroform:methanol (3:2, v/v). The resulting alcohol insoluble residues (AIRs) were air-dried overnight and stored for further use. AIRs were extracted sequentially with 2% (w/v) CaCl_2_, 3% (w/v) Na_2_CO_3_ and 4 M KOH. Samples were centrifuged between each extraction (1500*g*, 30 min) and the supernatants collected, dialysed (MWCO of 6–8 kDa) against distilled water and freeze-dried. KOH extracts were neutralised with glacial acetic acid prior to dialysis.

The *E*. *subulatus* samples used for microscopy were fixed for 1 h in seawater containing 4% paraformaldehyde and 10% glycerol. Samples were then washed twice in PBS before immunolabelling. Other algae used for microscopy were fixed overnight in PEM buffer (50 mM PIPES, 5 mM EGTA, 5 mM MgSO_4_, pH 6.9) containing 4% glutaraldehyde and 1% (w/v) caffeine. Samples were then washed twice in PBS before being dehydrated in an ethanol series of 5, 10, 15, 20, 30, 40, 50, 60, 70, 80, 90, and twice 100% at 4°C. Samples were then embedded in LR White resin, and polymerised at 37°C. Resin-embedded material was then sectioned (1 μm thickness). Fluorescence imaging of BAM probe binding to sections was achieved with indirect immunofluorescence labelling procedures with 5-fold dilutions of hybridoma supernatants followed by 100-fold dilution of anti-rat-IgG-whole molecule-FITC (Sigma-Aldrich, F1763) as described for other rat monoclonal probes [41]. Sections were stained prior to analysis with 0.1% (w/v) Toluidine Blue O in 0.2 M sodium phosphate buffer, pH 5.5.

## Results

### Comparative analysis of monoclonal antibody binding to brown algal polysaccharides

**Fig 1:**
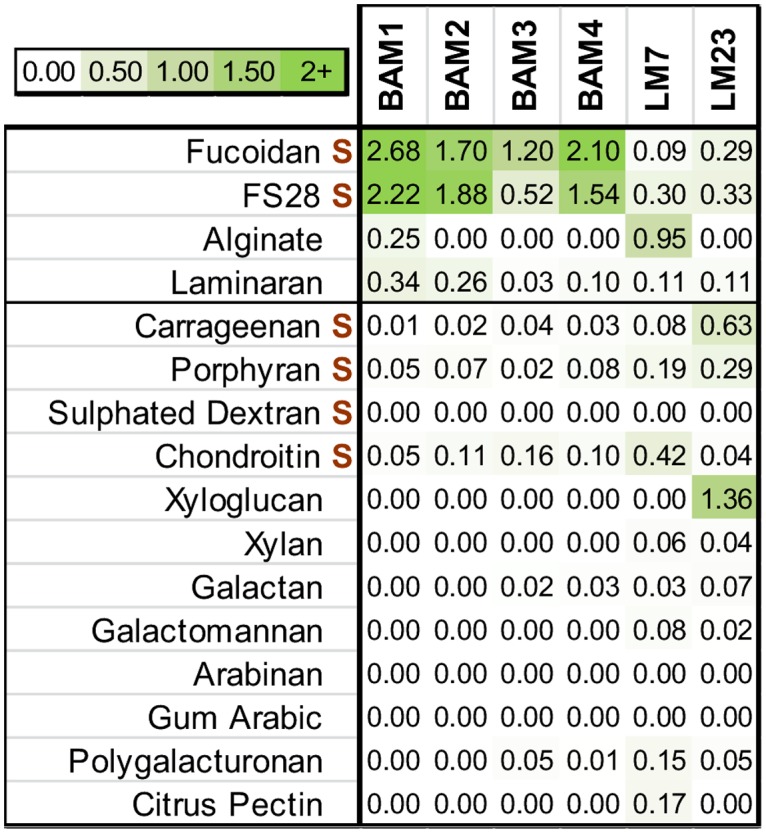
Heat map showing the relative binding of the BAM1 to BAM4, LM7 and LM23 MAbs to a range of brown algal, sulfated, red algal and land plant polysaccharides. Binding was determined by ELISA with 50 μg/ml of polysaccharide samples and 25-fold dilutions of antibody hybridoma supernatants. S indicates polysaccharides containing sulfate residues. The colour scale in relation to absorbance values is shown top left. Values shown are means of 4 replicates and in all cases standard deviations (SDs) were <0.1 absorbance units.

Four novel fucan-binding rat monoclonal antibodies (MAbs), designated Brown Alga Monoclonal antibodies (BAM), are described in this study. All were generated subsequent to immunization with commercially available fucoidan from *F*. *vesiculosus* (Sigma-Aldrich, F5631) coupled to BSA to generate an immunogen. Together with BAM1 to BAM4, two previously described antibodies were found to bind to cell wall materials derived from brown algae. MAb LM7 is directed to pectic homogalacturonan [42] but also binds to alginate, and cross-reactivity of alginate antibodies and pectin has previously been reported [43]. MAb LM23 binds to xylosyl residues in various glycans [44,45] and was found to also bind to brown algal cell wall materials. Analysis of the binding of all MAbs to the fucoidan used as the immunogen, to an isolated sulfated fucan FS28 [38], to brown algal polysaccharides alginate and laminaran, and to a range of other sulfated polysaccharides and polysaccharides from land plant cell walls is shown in Fig. 1. MAbs BAM1, BAM2 and BAM4 display a strong and specific binding to both fucan samples. MAb BAM3 shows a preference for the fucoidan sample over FS28 and, although at low antibody dilution it does not produce as high a signal as the other fucan-directed MAbs, it has a 50% of maximum signal titre that is >600. Titration of MAb binding to the fucan and alginate samples is shown in Fig. A in S1 File. Of the brown algal polysaccharides LM7 binds preferentially to the alginate sample and LM23 binds preferentially to the fucan samples albeit weakly. The impact of varying sodium chloride concentrations during antibody binding was assessed and increasing its concentration reduced binding a little for all four antibodies as shown in Fig. B in S1 File.

### The role of sulfate in BAM epitope recognition

**Fig 2:**
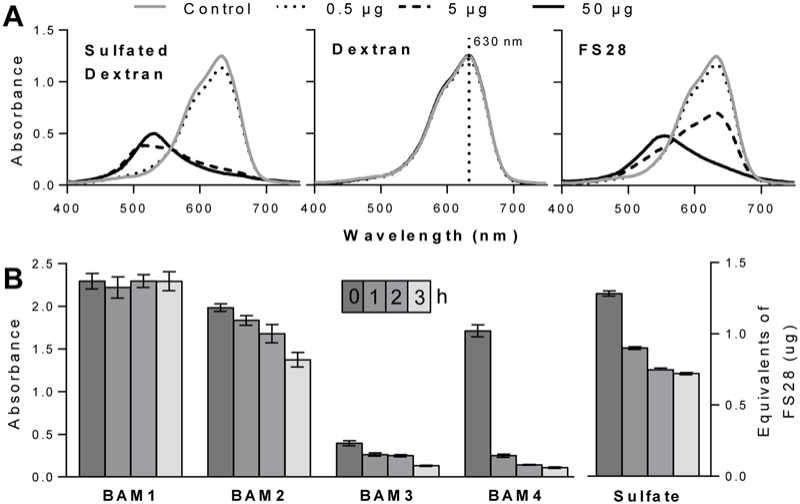
Sulfate assay and de-sulfation. (**A**) Azure A absorbance spectra changes in the presence of differing levels (0 to 50 μg) of sulfated dextran, dextran and FS28. (**B**) Effect of de-sulfation on BAM MAb recognition of FS28. Samples of FS28 were collected over 4 time points (0, 1, 2 and 3 h) during a de-sulfation treatment with chlorotrimethylsilane and assayed using ELISA. Absorbance values are means of 4 replicates and error bars indicate SD. Sulfate levels as equivalents to native FS28 (w/w).

Due to the potential for variation in the density and patterning of sulfate groups on the fucan family of polysaccharides, it was required to determine if any of the MAbs had a sulfate-requiring epitope. Azure A is a cationic dye capable of binding to sulfate residues on polysaccharides, and is widely used to measure the sulfate content of heparin [46]. Addition of a sulfated polysaccharide to an Azure A solution causes a colour change from blue to purple, as seen by sulfated dextran and FS28 (Fig. 2A). For comparison addition of a non-sulfated dextran has no effect on the spectrum of Azure A. Therefore, increasing sulfate levels can be measured by a decrease in absorbance at 630 nm.

The sulfated fucan fraction FS28 was de-sulfated using chlorotrimethylsilane (CTMS) in anhydrous pyridine [47]. Sulfate analysis revealed that the majority of de-sulfation occurred in the first hour of the reaction, with only small decreases occurring in the following 2 h (Fig. 2B). ELISA analysis of the samples for alterations of MAb binding revealed no change for BAM1 recognition and a slow decline for BAM2 and BAM3 over 3 h. In the case of BAM4 there was a large decrease in binding to FS28 (>80%) in the first hour of the de-sulfation procedure. These observations suggest that BAM1 binds to a non-sulfate containing epitope and that BAM4 binds to a sulfated epitope. The BAM2 and BAM3 epitope may require sulfate groups for recognition but it is also possible that the de-sulfation process results in other changes to glycan structures that impact on binding.

**Fig 3:**
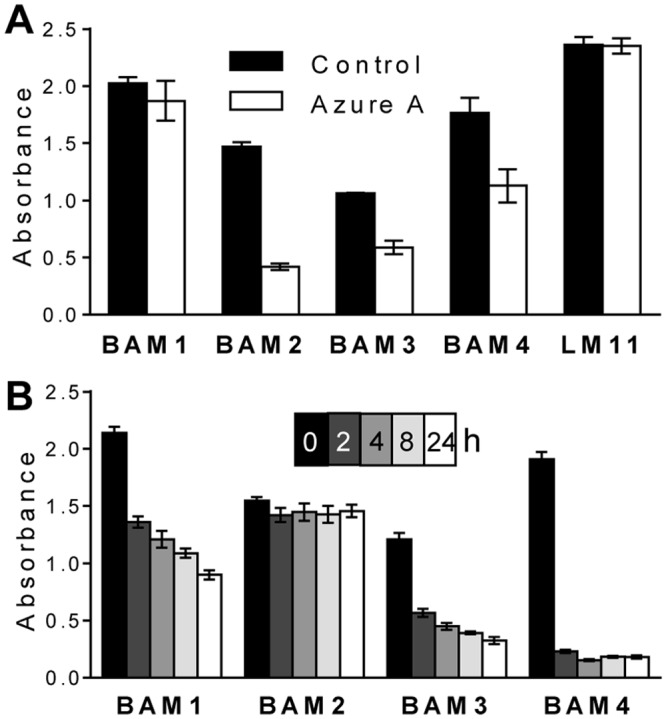
Effect of Azure A inhibition and sodium metaperiodate treatment on BAM antibody recognition of fucoidan. ELISA plates were coated with fucoidan and (**A**) pre-incubated with Azure A for 1 h, or equivalent control plates with buffer only, or (**B**) exposed to sodium metaperiodate for 0 to 24 h at 4°C in the dark. Absorbance values are means of 4 replicates and error bars indicate SD. LM11 binding to non-sulfated xylan was used as a control for Azure A inhibition.

To further explore the role of sulfate in epitope recognition the potential of the sulfate-binding dye Azure A to interfere with MAb recognition of the fucoidan sample was assessed. After coating and blocking of microtitre plates, Azure A (1 mg/ml) was incubated with the immobilised fucan for 1 h and plates were washed prior to the antibody incubation steps. As a control to assess non-specific inhibition by Azure A in MAb recognition of a non-sulfated glycan, Azure A was also incubated with oat spelt xylan prior to probing with xylan MAb LM11 [48]. The results shown in Fig. 3A indicate that BAM1 and LM11 binding to glycans was unaffected by Azure A whereas BAM4, BAM3 and BAM2 displayed inhibition of binding by the presence of the sulfate-binding dye, and the binding of BAM2 was reduced by over 70% (Fig. 3A). For BAM1 recognition, the non-inhibition by Azure A is consistent with the previous interpretation of BAM1 binding to a non-sulfate containing epitope. For BAM2, BAM3 and BAM4, clear conclusions cannot be made from this assay alone. Indeed, one interpretation could be that sulfate groups may be an element of the BAM2, BAM3, and BAM4 epitopes. However, the loss in BAM4 binding upon Azure A competition is not as strong as one may expect for a MAb recognizing a sulfated epitope (Fig. 2B). It is possible that Azure A and BAM4 might both compete for a sulfated epitope, leading to a lower apparent inhibition. On the other hand a sulfate group bound by Azure A and present in the close vicinity to BAM2 and BAM3 epitopes might also impair their binding ability, making it impossible to conclude from this assay alone if sulfate groups are elements of their epitopes.

Sodium metaperiodate is capable of cleaving the bond between two carbon atoms that both have associated hydroxyl groups (vicinal diols) and in homofucans these are present in fucose residues linked by 1,4-linkages, but not in the case of 1,3-linkages. Sodium metaperiodate sensitivity can be used to confirm antibody recognition of some glycan structures [39]. However, sulfate residues inhibit the action of sodium metaperiodate, due to the sulfation at the required OH groups [49]. Fucoidan-coated ELISA plates were treated for 2, 4, 8 and 24 h with sodium metaperiodate, and then the impact on the binding of the BAM MAbs determined. As shown in Fig. 3B the BAM4 epitope is the most susceptible to sodium metaperiodate treatment and this might be indicative of the presence of 1,4-glycosyl residues in the BAM4 epitope. Those of BAM1 and BAM3 display a moderate sensitivity and BAM2 is largely unaffected even after 24 h. The basis of periodate insensitivity in the latter case is not known, but it may be indicative of the presence of 1,3-glycosyl residues and/or of sulfate groups in the epitope, or alternatively of an epitope composed of residues other than fucose but with no vicinal diols.

We propose, based on the accumulated evidence outlined above, that the BAM1 MAb binds to an un-sulfated epitope present in sulfated fucans and that BAM4, with the highest sensitivity to de-sulfation, binds to a sulfate-containing epitope. BAM2 and BAM3 MAbs are specific to sulfated fucan/fucoidan preparations but their specificities in relation to the role of sulfation in their epitope structure are less clear. The panel of four BAM MAbs will therefore be useful for the study of fucan polymers in brown algal cell walls.

### Epitope detection chromatography analysis of sulfated fucan heterogeneity

**Fig 4:**
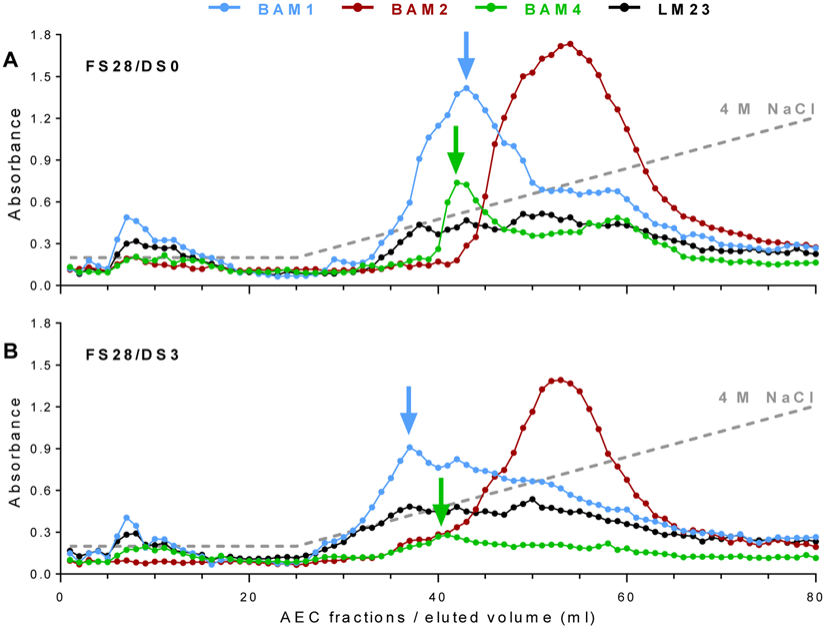
Epitope detection chromatographic (EDC) anion-exchange analysis. EDC analysis of A, sulfated (DS0) and B, de-sulfated (DS3) derivatives of FS28 using BAM1, BAM2, BAM4 and LM23 as detection tools. The elution gradient was 0 to 4 M NaCl from 26 ml to 80 ml elution volume. Blue arrows indicate shift in peak of BAM1 epitope elution associated with de-sulfation. Green arrows indicate loss of BAM4 epitope peak height but no shift in elution time. EDC profiles shown are representative of two chromatographic runs.

In order to further explore the heterogeneity of sulfated fucans, two extracts obtained from FS28, one with native sulfation (DS0) and the other produced by 3 h of de-sulfation (DS3), were analysed by epitope detection chromatography (EDC) [40] in anion-exchange mode. This chromatographic separation is based on charge, and fucans will be retained on the column and separated predominantly due to the number of sulfate residues and the presence of uronic acids which can also be abundantly found in fucan preparations. EDC involves the use of glycan MAbs as detection tools (using ELISAs) for the chromatographically-separated glycans. Both samples were run through an anion-exchange column, and fractions collected by applying a NaCl gradient. After their neutralisation, equivalent aliquots from the same chromatographic run were used to coat microtitre plate wells that were probed with MAbs BAM1, BAM2, BAM3, BAM4, LM7 and LM23. Both samples contained a small subset of detected BAM1 and LM23 epitopes that were not retained by the column (Fig. 4). The major epitope peaks were eluted in fractions 33 to 65 for DS0 and fractions 26 to 66 for DS3. The LM23 xylosyl epitope was detected at an equivalent level across these fractions, albeit eluting earlier in the DS3 sample, indicating little disruption to the xylosyl-containing domains in the fucan. In contrast, the BAM1, BAM2 and BAM4 epitopes were differentially detected in both extracts tested. The first BAM epitope to be detected in both samples after the initiation of the salt gradient (fraction 26) is the non-sulfate-requiring BAM1 that peaks in fraction 42 in DS0 and 37 in DS3. This confirms that the BAM1 epitope is carried by an acidic polymer and that de-sulfation reduces the number of charges on this polymer. In both profiles the BAM1 epitope is eluted in a range of separated peaks, indicating the heterogeneity in the charges of fucans carrying this epitope.

The BAM2 epitope is detected in later eluting fractions and peaks around fraction 54 in both FS28 extracts tested. The BAM2 EDC profiles are therefore complementary to the BAM1 patterns and indicate that the BAM1 and BAM2 epitopes are carried by less acidic and more acidic polymers respectively. In the de-sulfated sample the BAM2 peak is in the same fractions and shows little shift due to de-sulfation which may indicate an acidic but non-sulfated minor component of the preparation. In contrast the de-sulfation-sensitive epitope BAM4 was detected in two peaks in the DS0 sample, the less acidic broadly coinciding with that of the BAM1 epitope and also a late-eluting peak around fraction 58. The BAM4 epitope, as anticipated, shows the greatest loss in signal in DS3 compared to DS0, although it is detected across the fucan region of the chromatogram (Fig. 4). Equivalent EDC profiles of the BAM3 and LM7 epitopes, which only bind weakly to the samples, indicate recognition of the same regions of the chromatograms (Fig. C in S1 File).

Taken together the EDC experiments provide evidence that all four BAM epitopes are carried by molecules that are acidic. They support the conclusions that BAM1 binds to a non-sulfated epitope that is present on a sulfated glycan, and that BAM4 recognizes a sulfated epitope. BAM2 and BAM3 identified distinct and additional epitopes present in the fucoidan preparation but within polymers showing little elution shifts due to de-sulfation. These results also indicate that the FS28 fucan preparation contains a spectrum of molecules with highly variable patterns/densities of sulfate groups, and that aspects of this diversity can be assessed by the BAM MAbs.

### *In situ* fluorescence imaging of BAM epitopes reveals changes in cell-wall epitopes in relation to *Fucus vesiculosus* development

**Fig 5:**
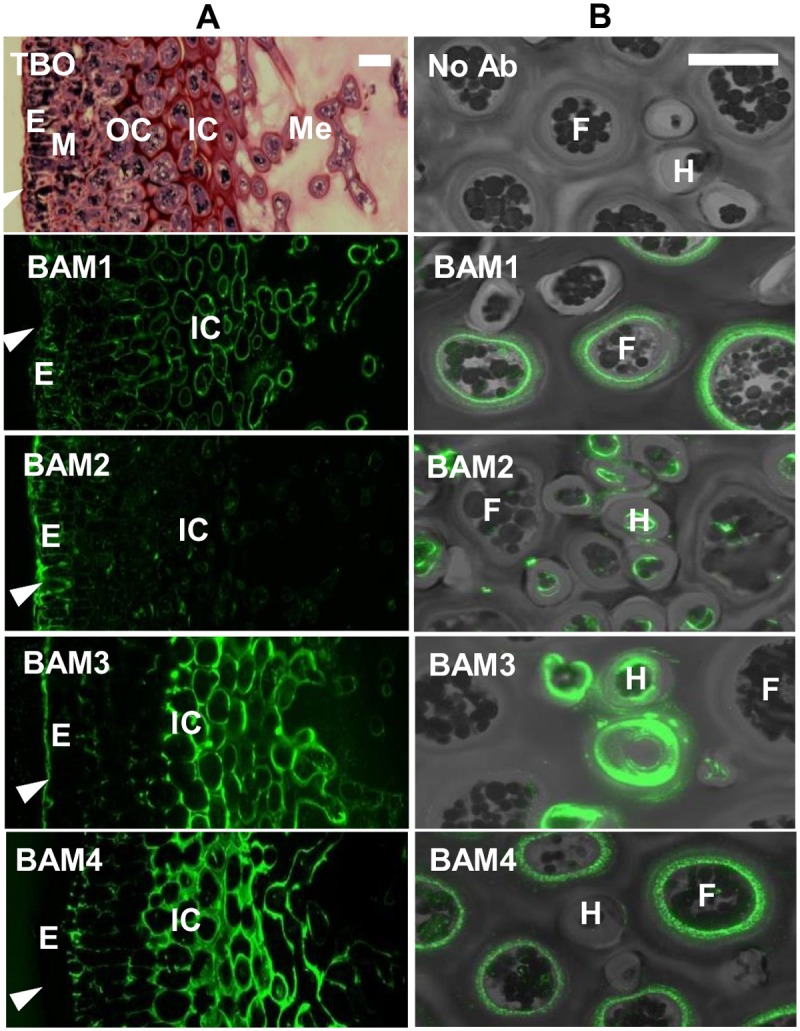
Indirect immunofluorescence analysis of *F*. *vesiculosus* receptacles. (**A**) Bright field image of Toluidine Blue O (TBO) stained resin-embedded section and fluorescence imaging of the BAM1 to BAM4 epitopes in equivalent sections. E, epidermis; M, meristoderm; OC, outer cortex; IC, Inner cortex; Me, Medulla. Arrowheads indicate outer surface. (**B**) Combined bright field/fluorescence of BAM epitopes in relation to hyphal cells (H) and filament cells (F) of the medulla region. Scale bars = 30 μm.

BAM1 to BAM4 MAbs were used in *in situ* fluorescence imaging procedures to ensure that the epitopes could be detected in intact cell walls and were not artefacts of fucan isolation. Outer regions of the reproductive receptacles of *F*. *vesiculosus* were selected for initial analysis as they allow easy visualisation of the epidermis, meristoderm, cortex and medulla (Fig. 5). In transverse sections it can be seen that the BAM1 epitope is detected uniformly in the cell walls of all tissues. The BAM2 epitope is detected around the epidermis and meristoderm, and was also present in intracellular structures. The BAM3 epitope was detected in the epidermis of the receptacles and in the inner cortex. The BAM4 epitope was not detected in the epidermis, neither nor in the meristoderm, but it was particularly abundant in cell walls and outer extracellular regions of the inner cortex (Fig. 5). This analysis indicates that the BAM epitopes have different locations within the *F*. *vesiculosus* receptacle, and thus suggests variations of the cell-wall structures including sulfated fucans across tissues. Examination of BAM probe binding to hyphal and filament cells in the medulla of receptacles where cells are less closely packed revealed detection of the BAM1 and BAM4 epitopes in the cell walls of the larger filament cells whereas the BAM2 and BAM3 epitopes were detected in the smaller hyphal cells (Fig. 5B).

**Fig 6:**
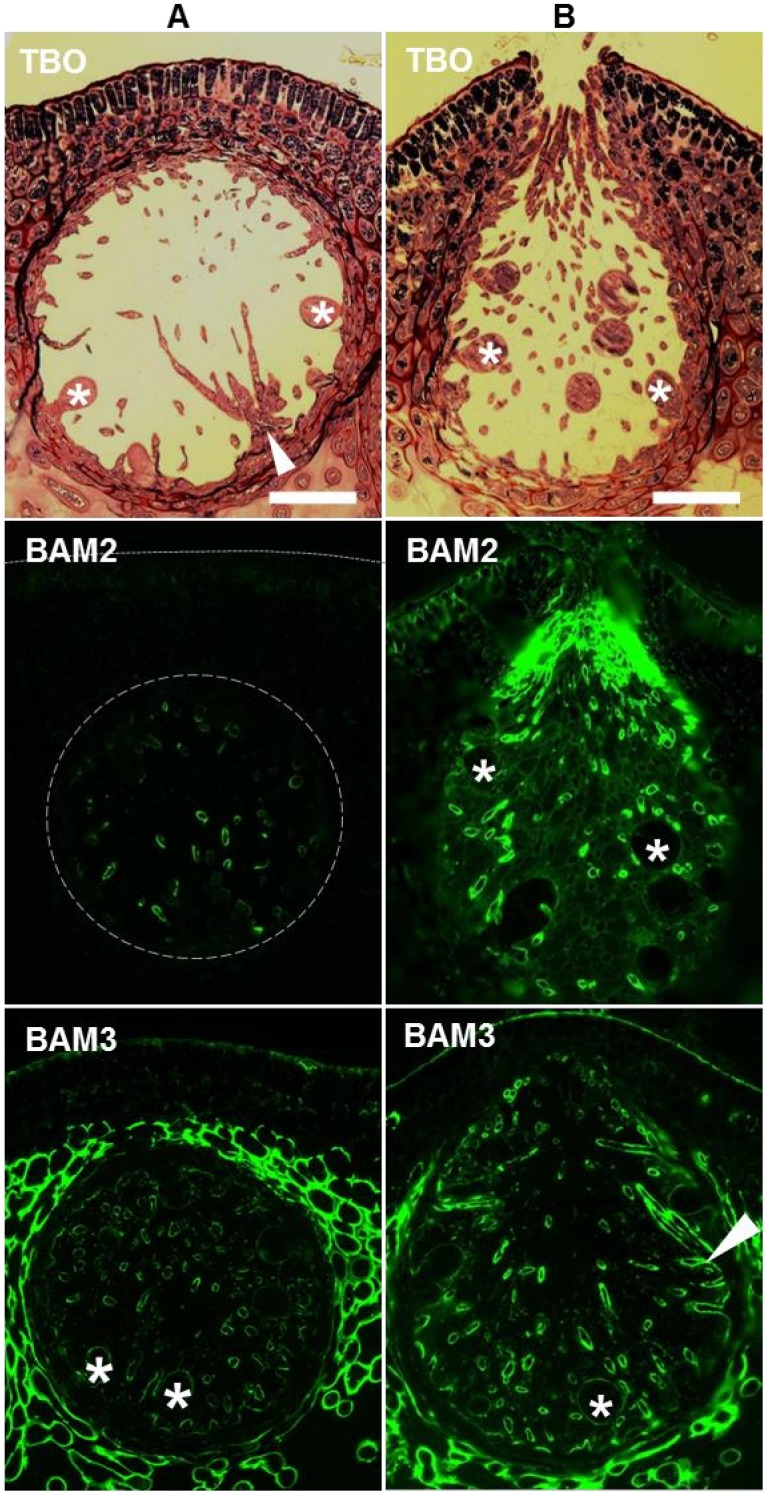
Indirect immunofluorescence analysis of *F*. *vesiculosus* female conceptacles. Bright field images of TBO-stained sections and fluorescence imaging of the BAM2 and BAM3 epitopes in equivalent sections. (**A**) Conceptacles prior to ostiole opening and (**B**) conceptacles after ostiole opening. Asterisks indicate oogonia and arrowheads indicate paraphyses. Scale bars = 100 μm.

*F*. *vesiculosus* is a dioecious species and the male (antheridia) and female (oogonia) reproductive structures are found in separate individuals. During development the conceptacle forms as a sphere which matures and releases the gametes through an opening or ostiole (Fig. 6). *In situ* fluorescence imaging with the MAbs across sections of reproductive receptacles of *F*. *vesiculosus* containing the female conceptacles provides contrasting images of unopened (no ostiole formed—spherical shaped) and opened conceptacles (including ostiole—teardrop shaped) after immunolabelling with BAM2 and BAM3 (Fig. 6). The BAM2 epitope is only weakly detected in paraphyses (sterile hairs) in unopened conceptacles, whereas after maturation of the conceptacle, it is detected in the meristoderm, and is also abundantly present inside the conceptacle around the paraphyses cells protruding from the ostiole. In contrast, the BAM3 epitope is detected strongly in cells surrounding the conceptacle and the oogonia in the conceptacle, but does not accumulate around the ostiole opening.

### *In situ* fluorescence imaging of the BAM4 epitope in *E*. *subulatus* reveals physiological changes in sulfate patterning during acclimation to salinity

**Fig 7:**
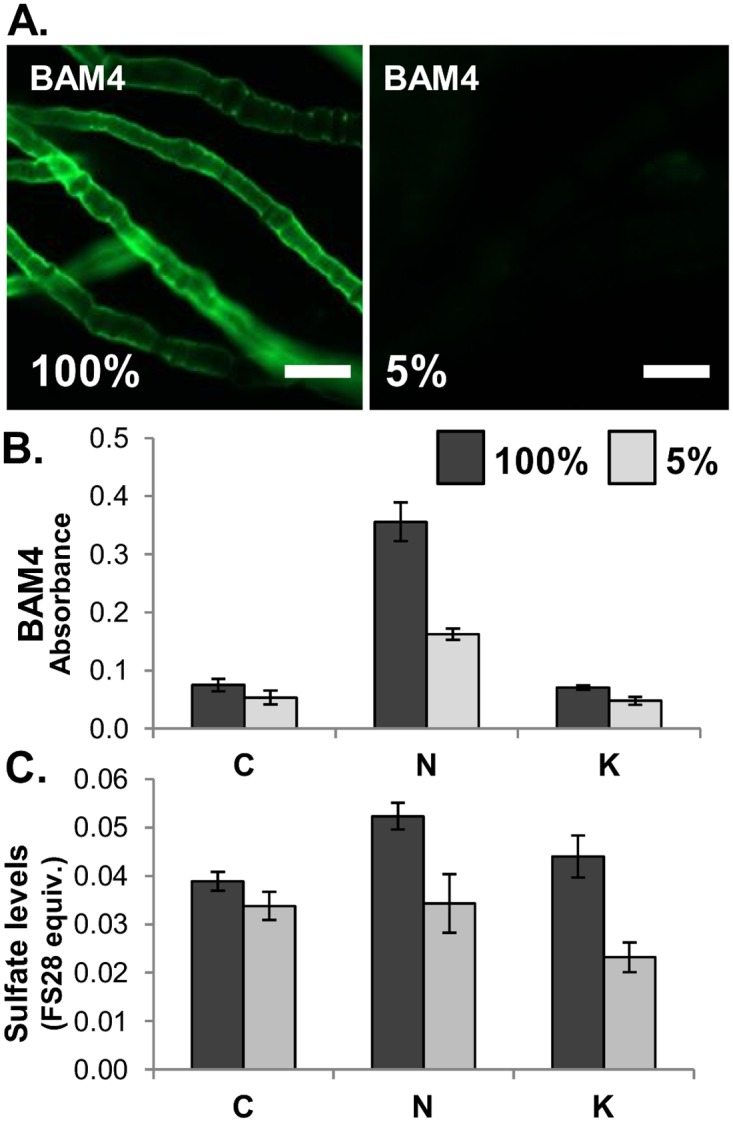
Analysis of *Ectocarpus subulatus*. Analysis of *E*. *subulatus* grown in seawater (100%) or in 20-fold diluted seawater (5%). (**A**) Indirect immunofluorescence detection of the BAM4 epitope at the surface of whole mount preparations, scale = 25 μm. (**B**) ELISA analysis of BAM4 epitope levels in extracts of AIR. C = CaCl_2_ extract, N = Na_2_CO_3_ extract, K = KOH extract. (**C**) Sulfate levels (FS28 equivalents w/w) in C, N and K extracts of AIR. Error bars indicate SD of 4 replicates.

The genus *Ectocarpus* groups several species of filamentous brown algae from environments exhibiting different levels of salinities. A strain of *E*. *subulatus* (Ec371), isolated from a freshwater environment, has been shown to be able to tolerate medium of various salinities [28]. Furthermore, acclimation of this strain to undiluted (100%) seawater (conductivity 4.8 S/m) or diluted (5%) seawater (conductivity 0.5 S/m) is associated with morphological changes. The BAM4 sulfated epitope was found to be the most abundant of the epitopes at the surface of intact, whole mount preparations of *E*. *subulatus*. *In situ* fluorescence imaging of the strain grown in 100% seawater indicates that the BAM4 epitope is present at the surface of most filaments, while it is not detected at the surface of the same strain when grown in 5% diluted seawater (Fig. 7A). Preparations of alcohol insoluble residues (AIRs) of *E*. *subulatus* grown in 100% and 5% seawater conditions were sequentially extracted with CaCl_2_, Na_2_CO_3_ and KOH to solubilize cell wall fractions for analysis of relative BAM4 epitope and sulfate levels. As shown in Fig. 7B, the BAM4 epitope was most abundantly detected in the Na_2_CO_3_ fractions with higher levels in the extracts for *E*. *subulatus* grown in 100% seawater. Sulfate levels, in terms of FS28 equivalents, were also higher in the 100% seawater samples with greater differentials between the 100% / 5% samples in the Na_2_CO_3_ and KOH extracts (Fig. 7C).

### Distribution of BAM sulfated fucan/fucoidan epitopes in brown algae

**Fig 8:**
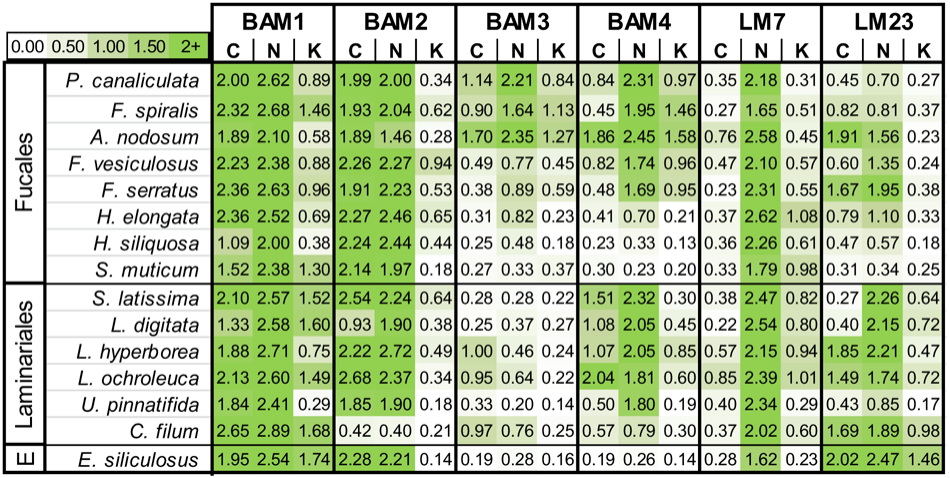
Heat map of antibody binding to cell wall extracts of a range of brown algae. Heat map of relative BAM1 to BAM4, LM7 and LM23 epitope levels as determined by ELISA absorbance obtained from 25-fold dilution of MAbs with 50 μg/ml of extracts from 8 species of the Fucales (F), 6 species of Laminariales (L) and 1 species of Ectocarpales (E). Fucale and Laminariale species are listed in relation to approximate occurrence from upper to lower regions of intertidal zones. Extracts of AIR with CaCl_2_ (C), Na_2_CO_3_ (N) and KOH (K) after dialysis and freeze-drying. The colour scale in relation to absorbance values is shown top left. Values shown are means of 4 replicates and in all cases SDs were <0.1 absorbance units.

To explore the distribution of the BAM sulfated fucan/fucoidan epitopes, and associated LM7 and LM23 epitopes, across an extended range of brown algae, samples were collected from eight species belonging to the Fucales (*Ascophyllum nodosum*, *Fucus serratus*, *F*. *spiralis*, *F*. *vesiculosus*, *Halidrys siliquosa*, *Himanthalia elongata*, *Pelvetia canaliculata*, *and Sargassum muticum*) and six species belonging to the Laminiarales (*Chorda filum*, *Laminaria digitata*, *L*. *hyperborea*, *L*. *ochroleuca*, *Saccharina latissima* and *Undaria pinnatifida*) in addition to an Ectocarpale, *Ectocarpus siliculosus*. Alcohol insoluble residues (AIRs) were prepared from whole algae and extracted sequentially as described above. Treatment with CaCl_2_ causes cross linking of the alginates rendering them insoluble, thus fractions obtained through this treatment should contain polysaccharides free from alginates. Extraction with Na_2_CO_3_ solubilises the alginate network and any associated polysaccharides, whilst 4 M KOH solubilise glycans more tightly bound within the cell walls. The relative abundances of the BAM1 to BAM4, LM7 and LM23 epitopes assessed by ELISA are shown in Fig. 8. All epitopes were present but at different levels in all the algal species examined. The BAM3 epitope was most abundant in three species of the Fucales. The BAM1 and BAM2 epitopes were consistently abundant in both the CaCl_2_ and Na_2_CO_3_ extracts, apart from the BAM2 epitope in *Chorda filum* extracts, whereas the BAM4 epitope occurred most often in the Na_2_CO_3_ extract along with alginates which are detected by LM7. The LM23 xylosyl epitope was most abundant in the CaCl_2_ and Na_2_CO_3_ extracts and showed wide variation in levels of detection contrasting with the BAM1 and BAM2 epitopes.

## Discussion

MAbs BAM1 to BAM4 all bind specifically to preparations of fucoidans and to brown algal cell walls *in situ*. Evidence indicates that BAM1 binds to a non-sulfated fucan epitope and that BAM4 binds to a sulfated fucan epitope. The role of sulfation in the BAM2 and BAM3 epitopes is not yet determined. The epitopes recognised by the MAbs are not yet entirely characterized and this will require future work with isolated oligosaccharides and enzymatic deconstructions. MAbs that can distinguish spatially and temporally distributed epitopes in cell walls of brown algae are useful tools for the dissection of polysaccharide structure, sulfation patterns and bioactivities in addition to their use in the study of cell wall architectures and functions.

### Use of MAbs in sulfated fucan/fucoidan analyses and potential of EDC

It is clear that the sulfated fucan/fucoidan group of glycans found in brown algae are highly heterogeneous in structural terms [16–20] and this adds a level of complexity to any assessment of the bioactivity of sulfated fucan preparations. The heterogeneity of a fucan preparation is directly addressed in the EDC analysis shown in Fig. 4, using three of the BAM MAbs. Acidic pectins extracted from land plant cell walls are readily eluted by <0.5 M NaCl [40], however, the FS28 fucan required in the region of 4 M NaCl for full elution from a similar anion-exchange column. In the process of this elution the BAM epitopes were differentially eluted, indicating a spectrum of acidic molecules with likely varying sulfation densities. The observation that the BAM2 epitope peak was not altered in mobility after partial de-sulfation is suggestive of a core component with a high acidic group density that is largely unaffected by the de-sulfation procedure used. In contrast, following de-sulfation, the earlier elution of the unsulfated BAM1 epitope, which co-eluted with the BAM4 epitope, suggests a less acidic component that is more sensitive to the de-sulfation process. Analysis of epitope presence in cell walls and tissues—discussed below—clearly indicates that epitopes can occur on separate molecules—as for example the differential occurrence of the BAM1/BAM4 epitopes in filament cells and the BAM2/BAM3 epitopes in hyphal cells of *F*. *vesiculosus* receptacles (Fig. 5). Such analytical approaches to dissect the distinct polymers that occur within a fucan preparation have considerable potential to directly address issues of heterogeneity and to elucidate the spectrum of molecules within a preparation or extract that is being applied to a particular end use. Moreover, it should also be possible using epitope tagging/affinity techniques to isolate specific sub-fractions of sulfated fucans for assessments of both structures and specific bioactivities. Potential links between particular oligosaccharide structural features/epitopes within molecules, for example the LM23 xylosyl epitope and the BAM epitopes, could also be explored by enzymatic interventions prior to EDC analyses or sandwich ELISA approaches [50].

### Use of MAbs in developmental and ecophysiological studies

In addition to the capacities of the BAM antibodies to discriminate components of fucan preparations, the BAM epitopes are clearly differentially regulated *in situ*. Modulations in epitope levels are observed during development of *F*. *vesiculosus* and in response to changes in salinity for *E*. *subulatus*.

Distinct cell morphologies are found in the tissue zones of *F*. *vesiculosus* receptacles. Cells in the medulla have heavily thickened walls and are further interspaced by an abundant intercellular matrix. Cell walls in the outer cortex and epidermis remain rather thin, except the epidermis outer wall. The four BAM epitopes have different patterns of detection across these tissues with the BAM2 and BAM3 epitopes being evident at the outer surface of the organ, and the BAM1/BAM4 epitopes being absent from this region (Fig. 5). The BAM2 epitope is mostly restricted to the thallus surface, in direct contact with the external environment. At this location cell wall components may act as an ionic barrier important for the osmotic adjustment during changes in salinity. It has been proposed [51–52] that cells are shielded by fucans of increasing sulfation toward the outer walls. The abundance of the BAM2 epitope (carried by the most acidic fraction of FS28) at the ostiole opening of *F*. *vesiculosus* female conceptacles (Fig. 6) may also reflect a role of a specific form of fucoidan in algal cell interactions with saline environments.

Salinity levels in coastal habitats can vary greatly for short periods of time due to tides, rainwater inputs or evaporation of tidal pools. Sudden changes in salt concentrations require that organisms in such habitats adjust their osmotic potential. Previous studies have pointed out that cell walls/extracellular matrices may contribute to cell ionic regulation by means of cation-binding to the negatively-charged polysaccharides, notably sulfated fucans in brown algae [21,51]. The *E*. *subulatus* Ec371 strain can grow in the laboratory under a broad range of salinities with all cells in its filamentous structure in contact with the environment [28]. It is of interest that the de-sulfation sensitive BAM4 epitope was the most abundant at the surface of intact, whole mount preparations of *E*. *subulatus* when grown in 100% seawater but not detected when grown in 5% seawater. This responsiveness to variations in salinity was also confirmed by assessment of total BAM4 epitope levels (Fig. 7). This difference demonstrates that the sulfation patterning at the surface of this brown alga can be actively regulated in relation to external salt concentrations. These cellular observations correlate well with transcriptomic analysis of genes encoding sulfotransferases (for the de-novo sulfated fucan assembly) which were induced under growth of *E*. *subulatus* in 100% seawater while the expression of genes coding for sulfatases involved in de-sulfation was reduced under low salinity [28]. These results further support the idea that sulfation density in cell walls, which would directly impact on the ion composition and cation-binding activity, would influence water potential in the vicinity of cell membranes and be an important factor in the osmotic adjustment to salinity stress.

The mapping of the occurrence of the four BAM epitopes across 15 species of brown algae (Fig. 8) demonstrates the wide abundance of the BAM1 and BAM2 epitopes in both the calcium chloride and sodium carbonate extracts, indicating that the carrying polymers are held within cell wall structures by a range of mechanisms. It is of interest that the BAM3 and BAM4 epitopes are more varied in distribution between species and taxonomic groups. The Fucales have a characteristic distribution of species in intertidal zones, ranging from *Pelvetia canaliculata* at the upper limit, to *Sargassum muticum* at the lower limit of the intertidal zone. Organising the species in this order (Fig. 8) shows the trend of increased detection of the BAM3 and BAM4 epitopes in extracts of Fucales in relation to algal zonation in the intertidal zone. The BAM3 epitope is most abundant in three Fucale species: *Pelvetia canaliculata*, *F*. *spiralis* and *Ascophyllum nodosum* and least abundant in brown algae which are always submerged such as *Sargassum muticum*. The Laminariales also have a distribution of species from the low water mark into the sublittoral zone; however, this distribution depends greatly on the exposure of the shore to wave action and the optical clarity of water. Hence these species are harder to order in this manner. The BAM3 epitope is less abundant in this group whereas the BAM4 epitope has a similar detection pattern in the Fucales and Laminariales with a low abundance in *Chorda filum*. Alginate (LM7) is consistently detected in the sodium carbonate fraction in all species, and a highly varied pattern of occurrence is seen for the LM23 xylosyl epitope. It remains to be elucidated how xylosyl-containing structural elements are arranged or linked to sub-fractions of the sulfated fucan group of polymers.

## Conclusion

The MAbs described in the present study are highly versatile molecular tools for the analysis and dissection of fucoidan preparations, including sulfated fucan polysaccharides. They also have the potential to generate defined sub-fractions in relation to anionic charges/sulfation which may be of importance in industrial applications. MAb use in conjunction with molecular tools such as defined oligosaccharides and enzymes directed to sulfated fucans will allow more precision in defining epitope and glycan structures. Moreover, the MAbs will be important molecular tools to integrate with cell biological, genomic and genetic approaches to understand the physiological and developmental mechanisms of brown algae.

## Supporting Information

S1 File Contains the following files: Fig. A. ELISA analysis of the four sulfated fucan-directed MAbs (BAM1, BAM2, BAM3 and BAM4), alginate-directed LM7, and xylosyl residue-directed LM23, binding to fucoidan, sulfated fucan FS28 and alginate. Polymers were coated on microtitre plates at 50 μg/ml and probed with a 5-fold dilution series of MAb hybridoma cell culture supernatants. Results shown are means of 4 replicates and SD are in all cases <0.1 absorbance units. **Fig. B**. ELISA analysis of the four sulfated fucan-directed MAbs (BAM1, BAM2, BAM3 and BAM4) binding to fucoidan in the presence of varying NaCl concentrations in phosphate buffer. The 137 mM NaCl concentration is equivalent to the PBS used for antibody experiments elsewhere in the study. Error bars indicate SD of 4 replicates. **Fig. C**. Epitope detection chromatographic (EDC) anion-exchange analysis of sulfated (DS0) and de-sulfated (DS3) derivatives of FS28 using BAM3, and LM7 as detection tools. Elution gradient was 0 to 4 M NaCl from 26 ml to 80 ml elution volume. EDC profiles shown are representative of two chromatographic runs.
(PDF)
