# Supplementary material for: Monoclonal Antibodies Directed to Fucoidan Preparations from Brown Algae
Source: PLoS One. 2015 Feb 18;10(2):e0118366. doi: 10.1371/journal.pone.0118366 (PMC4333822; doi:10.1371/journal.pone.0118366)
Supplement: S1 File — ELISA analysis of the four sulfated fucan-directed MAbs (BAM1, BAM2, BAM3 and BAM4), alginate-directed LM7, and xylosyl residue-directed LM23, binding to fucoidan, sulfated fucan FS28 and alginate. Polymers were coated on microtitre plates at 50 μg/ml and probed with a 5-fold dilution series of MAb hybridoma cell culture supernatants. Results shown are means of 4 replicates and SD are in all cases <0.1 absorbance units. Fig. B. ELISA analysis of the four sulfated fucan-directed MAbs (BAM1, BAM2, BAM3 and BAM4) binding to fucoidan in the presence of varying NaCl concentrations in phosphate buffer. The 137 mM NaCl concentration is equivalent to the PBS used for antibody experiments elsewhere in the study. Error bars indicate SD of 4 replicates. Fig. C. Epitope detection chromatographic (EDC) anion-exchange analysis of sulfated (DS0) and de-sulfated (DS3) derivatives of FS28 using BAM3, and LM7 as detection tools. Elution gradient was 0 to 4 M NaCl from 26 ml to 80 ml elution volume. EDC profiles shown are representative of two chromatographic runs. (PDF) [file pone.0118366.s001.pdf]

Supporting Information S1 File

Torode *et al*. ‘Monoclonal antibodies directed to fucoidan preparations from brown algae’

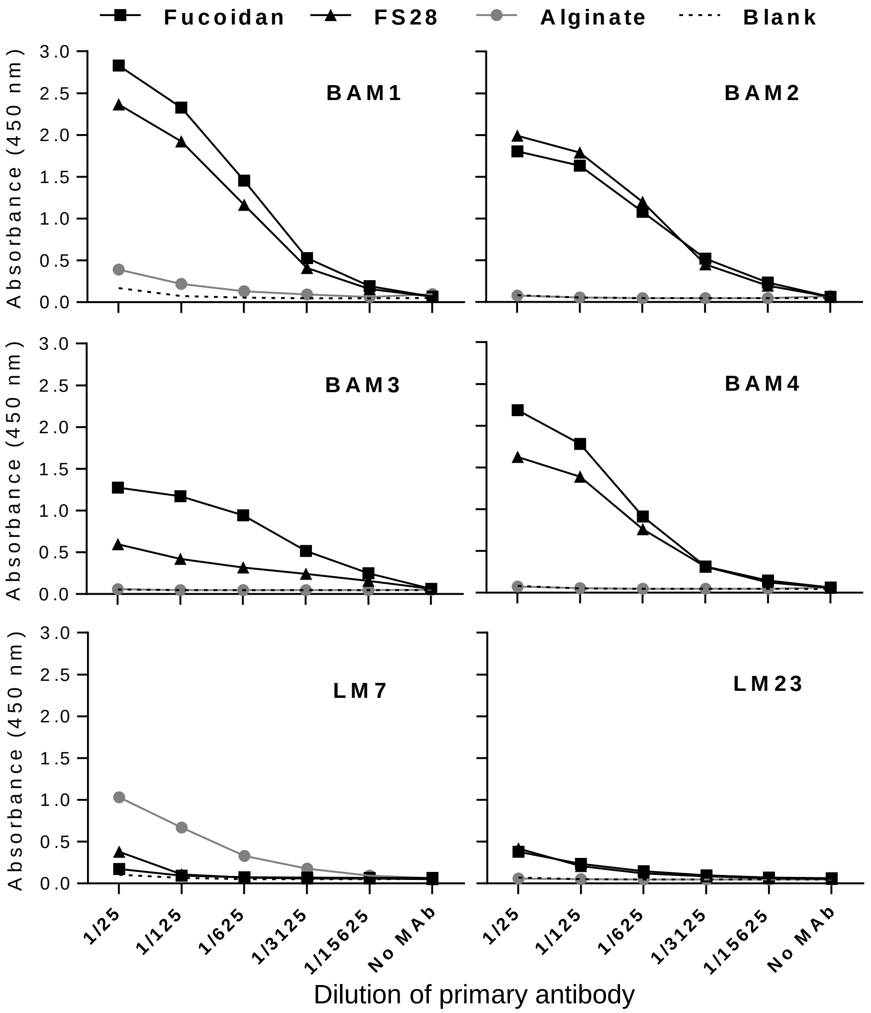

Figure A. ELISA analysis of the four sulfated fucan-directed MAbs (BAM1, BAM2, BAM3 and BAM4), alginate-directed LM7, and xylosyl residue-directed LM23, binding to fucoidan, sulfated fucan FS28 and alginate. Polymers were coated on microtitre plates at 50 µg/ml and probed with a 5-fold dilution series of MAb hybridoma cell culture supernatants. Results shown are means of 4 replicates and SD are in all cases <0.1 absorbance units.

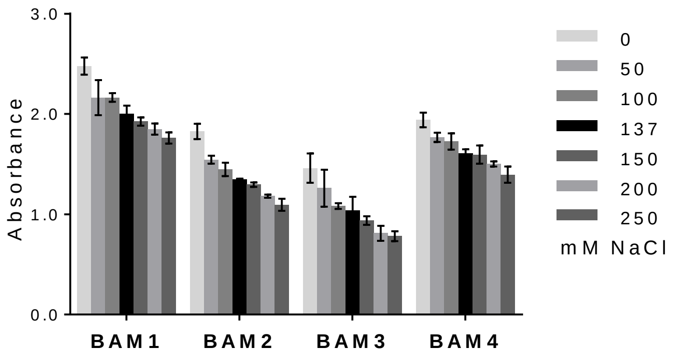

Figure B. ELISA analysis of the four sulfated fucan-directed MAbs (BAM1, BAM2, BAM3 and BAM4) binding to fucoidan in the presence of varying NaCl concentrations in phosphate buffer. The 137 mM NaCl concentration is equivalent to the PBS used for antibody experiments elsewhere in the study. Error bars indicate SD of 4 replicates.

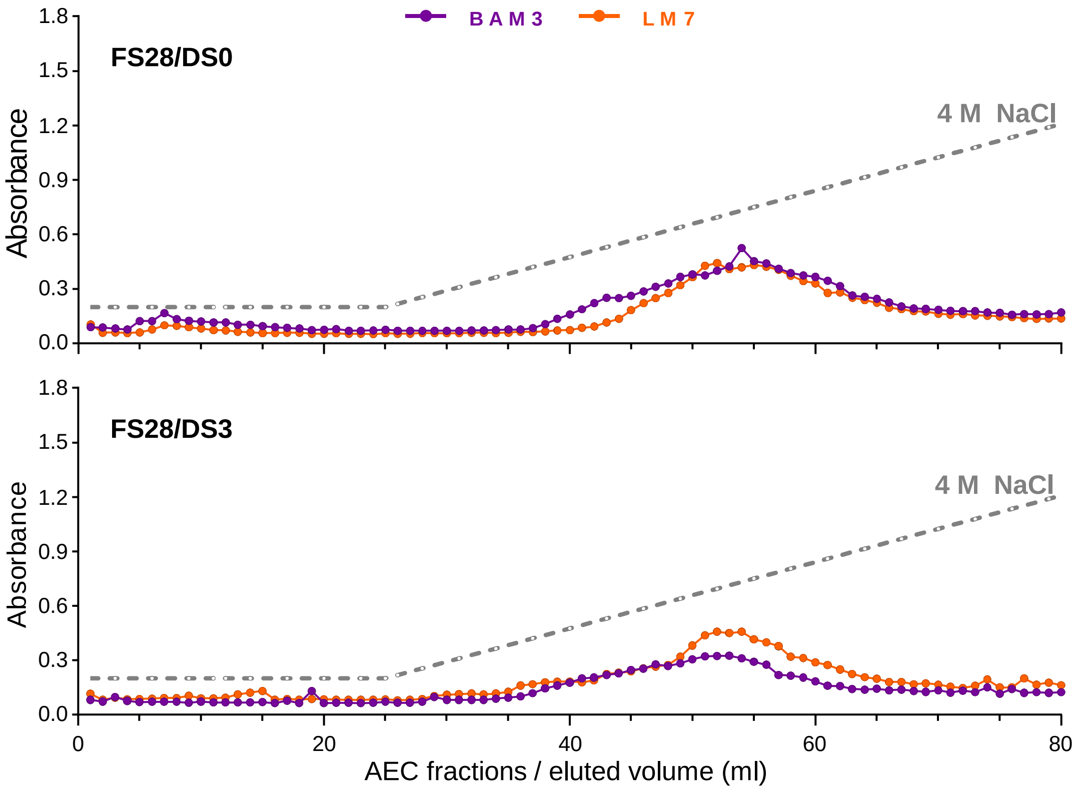

Figure C. Epitope detection chromatographic (EDC) anion-exchange analysis of sulfated (DS0) and de-sulfated (DS3) derivatives of FS28 using BAM3 and LM7 as detection tools. Elution gradient was 0 to 4 M NaCl from 26 ml to 80 ml elution volume. EDC profiles shown are representative of two chromatographic runs.
